# Study of Coxsackie B viruses interactions with Coxsackie Adenovirus receptor and Decay-Accelerating Factor using Human CaCo-2 cell line

**DOI:** 10.1186/1423-0127-21-50

**Published:** 2014-05-21

**Authors:** Samira Riabi, Rafik Harrath, Imed Gaaloul, Lamjed Bouslama, Dorsaf Nasri, Mahjoub Aouni, Sylvie Pillet, Bruno Pozzetto

**Affiliations:** 1 Laboratory of Transmissible Diseases LR99-ES27, Faculty of Pharmacy, Avenue Avicenne 5000, Monastir, Tunisia; 2 Laboratory of Bacteriology-Virology, GIMAP EA3064, Faculty of Medicine of Saint-Etienne, Saint-Etienne, France; 3 Laboratory of Bio-Active Substances, Center of Biotechnology of Borj Cedria, Borj Cedria, Tunisia; 4 Laboratory of Bacteriology-Virology-Hygiene, University Hospital Saint-Etienne, Saint-Etienne, France

**Keywords:** CV- B, CaCo-2 cell line, Receptors, Phenotypes, Variants, TEER

## Abstract

**Background:**

Decay Accelerating Factor (DAF) and Coxsackievirus-Adenovirus Receptor (CAR) have been identified as cellular receptors for Coxsackie B viruses (CV-B). The aim of this study is to elucidate the different binding properties of CV-B serotypes and to find out if there are any amino acid changes that could be associated to the different phenotypes.

Twenty clinical CV-B isolates were tested on CaCo-2 cell line using anti-DAF (BRIC216) and anti-CAR (RmcB) antibodies. CV-B3 Nancy prototype strain and a recombinant strain (Rec, CV-B3/B4) were tested in parallel. The P1 genomic region of 12 CV-B isolates from different serotypes was sequenced and the Trans-Epithelial Electrical Resistance (TEER) along with the virus growth cycle was measured.

**Results:**

Infectivity assays revealed clear differences between CV-B isolates with regard to their interactions with DAF and CAR. All tested CV-B isolates showed an absolute requirement for CAR but varied in their binding to DAF. We also reported that for some isolates of CV-B, DAF attachment was not adapted. Genetic analysis of the P1 region detected multiple differences in the deduced amino acid sequences.

**Conclusion:**

Within a given serotype, variations exist in the capacity of virus isolates to bind to specific receptors, and variants with different additional ligands may arise during infection in humans as well as in tissue culture.

## Background

The six serotypes of coxsackie B viruses (CVB1- 6) are members of the Human enterovirus B (HEV-B) species of the Enterovirus genus within the *Picornaviridae* family. They are causative agents of a broad spectrum of clinically relevant diseases including acute and chronic myocarditis, meningitis and possibly autoimmune diabetes [1-3]. The 7.4 kb positive stranded RNA genome of CV-B consists of a 5_untranslanslated region (5_UTR) followed by a single polyprotein coding region and a 3_UTR, flanked by a poly A-tail [4]. The first part of the polyprotein (P1) encodes the four capsid proteins, and the second and third part of the polyprotein (P2 and P3, respectively) encode non-structural proteins involved in genome processing and RNA synthesis [5]. The four capsid proteins, VP1–VP4, assemble into a pseudo -T = 3 icosahedral capsid. The VP1–VP3 make up the outer surface of the viral particle, while VP4 is embedded within the inner surface of the capsid [5]. A prominent feature of the capsid surface is a small depression surrounding the fivefold axis, the so-called “canyon” which is proposed to enable virus attachment by interaction with cell surface molecules [6,7]. Receptor binding induces conformational changes which facilitate the release of viral RNA into host cells [8,9].

The identification of specific cellular receptors and viral receptor-binding sites are among the major goals of fundamental virology. To date, two types of cellular molecules have been identified as cell surface receptors for CV-B. CAR is a 46 kDa membrane glycoprotein and part of a larger protein complex in the tight junction of the cell and might function as a cell–cell adhesion molecule [10-12]. In both polarized cells and mucosal epithelium, the CAR protein is absent from the apical surface and is localized to intercellular tight junctions [13,14]. CAR-negative and nonpolarized cells are considered to be non permissive for CV-B infection in vitro. Additionally, CV-B serotypes 1, 3, and 5 have been found to bind Decay-Accelerating Factor (DAF/CD55) as a co-receptor [9,15-17]. DAF, a 70 kDa glycosylphosphatidylinositol-anchored membrane protein, is a member of the regulators of complement activation (RCA) family that regulate complement activation by binding to and accelerating the decay of convertases, the central amplification enzymes of the complement cascade [18]. DAF functional region consists of four short consensus repeats (SCR1 to 4) [16,17,19]. This protein was also described as a receptor for echoviruses, Enterovirus 70, and Coxsackievirus A 21 [20-22]. Although DAF binding is likely to facilitate viral adsorption and mediate tropism, the availability of DAF receptor molecules on the host seems to be insufficient to facilitate cell entry and lytic infection of CV-B even to the DAF-adapted strains [16,17,23]. Upon transfection with CAR cDNA, non-infectable hamster CHO cells become susceptible to infection with CV-B [24,25]. Moreover, even CV-B strains with strong DAF-binding properties require the CAR protein to mediate lytic infection [23-26]. Therefore, it appears that DAF and CAR capacities to impart permissiveness to infection are not equivalent. Virus interaction with CAR, but not with DAF, leads to a post attachment event that is essential for infection to proceed. During eclipse, enterovirus capsids undergo conformational changes that lead to the release of viral RNA into the cytoplasm [27]. After attachment, most cell-associated viruses are converted into an irreversibly altered form, the A particle, which has lost the internal capsid protein VP4, and no longer interacts with cellular receptors or infects receptor-bearing cells [28].

With regard to these findings, the current study was performed to elucidate the CV-B interactions with CAR and DAF receptors on the cell surface. To address this topic, clinical CV-B isolates of different serotypes were selected to assess the different binding properties and receptor usage in CaCo-2 cells, a well-characterized CAR-positive and DAF positive polarized cell line with apical and basolateral surfaces. In addition, Trans-Epithelial Electrical Resistance (TEER) measurement, which is indicative of cell integrity and works using the principle that well-formed junctions and healthy membranes have a higher electrical resistance, was carried out. The molecular basis of different binding patterns was also investigated.

## Methods

### Cell lines

CaCo-2 Cell line (human colon adenocarcinoma cell line) was kindly provided by the department of Immunity of mucous membranes and pathogenic agents (GIMAP, Faculty of medicine Jacques Lisfranc, Saint-Etienne, France). These cells were cultured in Dulbecco’s Modified Eagle medium (DMEM-F12) (PAA Laboratories) supplemented with 10% of Fetal Bovine Serum (FBS) (GIBCO) and antibiotic antimycotic solution 1× (Sigma) containing 100 units/ml penicillin, 100 μg/ml streptomycin and 25 μg/ml ciprofloxacin.

KB cell line (human squamous carcinoma cell line) was grown in RPMI 1640 medium (Sigma) containing 10% of Fetal Bovine Serum, L-glutamine (2 mM; Sigma) and the same antibiotics. KB cells were used for virus quantification using the TCID_50_ (tissue culture infectious dose 50%) method [29] and as controls in the TEER measurements. The cells were incubated at 37°C in an atmosphere of 5% CO2.

### Viruses

Twenty CV-B clinical isolates of different serotypes (CV-B1 à 6): **CV-B1** (CV-B1#032, CV-B1#0807, CV-B1#0609), **CV-B2** (CV-B2#N, CV-B2#37222, CV-B2#0610 M, CV-B2#2000), **CV-B3** (CV-B3#37335, CV-B3#523-21, CV-B3#02), **CV-B4** (CV-B4#N,CV-B4#37428, CV-B4#07, CV-B4#0612 and CV-B4-JVB), **CV-B5** (CV-B5#37534, CV-B5#2003, CV-B5#99, CV-B5#2000) and **CV-B6** (CV-B6#041) were used. CV-B3 Nancy prototype strain and a recombinant strain (Rec, CV-B3/B4, provided by the Virology Department (GIMAP EA3064) of the Faculty of Medicine of Saint Etienne, France) were tested in parallel. The Rec strain is the first enterovirus derived from interserotypic recombination event occurring in the VP3 coding region and leading to a chimeric CV-B3/CV-B4 type. Four subsequent propagations of viruses were performed in KB cells in a serum-free medium.

### Antibodies

For CAR detection, a monoclonal IgG1 antibody, RmcB (AbCys S.A.) was used. DAF detection was performed using commercially purified mAb, BRIC216 (AbCys S.A), a mouse anti-SCR3 (short consensus repeat 3 of DAF). The amounts of anti-CAR and anti-DAF required to inhibit infection of CaCo-2 cells were titrated by pre-incubation of CV-B-CaCo-2 cells with different concentrations of each antibody from 2 to 10 μg.

### Trans-Epithelial Electrical Resistance (TEER) measurement

To establish polarized monolayers, Caco-2 colonic epithelial cells were cultured in (DMEM)-F12 (PAA laboratories) with 10% of Fetal Bovine Serum (GIBCO) on polyester tissue culture inserts (Costar Transwell Clears; 12-mm diameter, 0.4-μm pore size) until TEER measurement with an epithelial voltohmmeter (EVOM; World Precision Instruments, Inc.) was stable (300 to 350 Ω.cm^2^ in 12 to 14 days). For the passage of viruses on CaCo-2 cell line, cells were infected with viruses at MOI = 1 for 1 h at room temperature. The monolayer was then washed and incubated at 37°C. The epithelial voltohmmeter was then used to follow, at various post-inoculation times (pi), the variation of TEER of CaCo-2 cells along with the viral growth cycle. The TEER measurements were performed first every 30 min and then every hour until 24 h. KB cells were used as controls in this assay.

### Infection and inhibition assays

CaCo-2 cells were seeded in 96-well plates. Each plate was pre-treated for 2 h at 37°C with 5 μg of RmcB, BRIC216 or a combination of both antibodies. The antibody concentrations used were titrated prior to inhibition analyses. The wells were washed twice with 1× PBS and viruses (MOI = 0.01) were used to infect the 60% confluent monolayers. CaCo-2 cells infected in the absence of blocking antibodies are used as control. 200 μl serum-free medium were added and the cells were controlled 48 h post infection for a possible cytopathic effect. 50 μl aliquots were also removed 48 h post infection and TCID_50_ was determined on KB cells.

### RNA extraction and reverse transcription

Viral RNA was extracted from 100 μl of viral supernatant using a QIAamp viral RNA mini kit (QIAGEN, France) according to the manufacturer’s recommendations. Ten microliters of extracted RNA were reverse-transcribed into cDNA at 42°C for 45 min using 200 units of SuperScriptIII reverse transcriptase and 2.5 ng/μl of random primers (Invitrogen, Cergy Pontoise, France) in the presence of 10 units of RnaseOUT recombinant RNase inhibitor (Invitrogen, Cergy Pontoise, France).

### Amplification experiments

All the amplification experiments were performed in a Mastercycler gradient thermal cycler (Eppendorf, Hamburg, Germany).

i. **VP1 region.** Five microliters of cDNA were amplified using 50 pmol of the 292 and 222 primers (Table 1) and 1.25 units of Platinum *Taq* DNA polymerase (Invitrogen, Cergy Pontoise, France) in 50 μl of reaction mixture according to the protocol described by Oberste et al. [30]. A band of the expected size of 357 bp was observed after agarose gel electrophoresis.

ii. **VP2 and VP4 regions.** Five microliters of cDNA were amplified using 80 pmol of each primer (Table 1) [31] and 1.25 units of Platinum *Taq* DNA polymerase (Invitrogen, Cergy Pontoise, France) in 50 μl of reaction mixture. Some minor modifications were made to the amplification program used by Nasri et al. (2007) and included an initial cycle of 95°C for 5 min, 39 further cycles of denaturation at 95°C for 45 s, annealing at 48°C for 45 s (raised to 50°C for VP4 region), extension at 72°C for 45 s and a final extension cycle at 72°C for 10 min. The bands of the expected size of 584 bp and 583 pb were respectively observed after electrophoresis in agarose gel.

iii. **VP3 and VP1-2A regions.** Five microliters of cDNA were amplified using 80 pmol of each primer (Table 1) and 1.25 units of Platinum *Taq* DNA polymerase (Invitrogen,Cergy Pontoise, France) in 50 μl of reaction mixture. The amplification included an initial cycle of 95°C for 5 min; 40 further cycles of denaturation at 94°C for 30 s, annealing at 48°C for 45 s, extension at 72°C for 90 s and a final extension cycle at 72°C for 5 min. The bands of the expected size of 1142 bp and 904 bp were observed respectively. For VP1-2A region, alternative amplification experiments were performed using 80 pmol of the RC5M or RC5 (sense) and RC10 or RC11 and RC12 primers (antisense) (Table 1). The amplification includes the same program of cycles as VP2 region generating a band of 596 pb (using RC5M and RC10) or 600 pb (using RC5/RC11 or RC5/RC12) as expected.

**Table 1:** Primers used for the amplification of the P1 region

**Region**	**Primer**	**Orientation**	**Sequence (5’-3’)**^***a***^	**Position**^***b***^	**Reference**
**VP1**	292	Sense	MIGCIGYIGARACNGG	2613–2628	30
	222	Antisense	CICCIGGIGGIAYRWACAT	2969–2951	
**VP2**	AM12	Sense	GARGARTGYGGITAYAGYGA	962–981	31
	AM32	Antisense	TTDATCCAYTGRTGIGG	1545–1529	
**VP3**	RC7	Sense	GTVHDIAAYGCIGGHATGGG	1463-1482	This study
	RC8	Antisense	GTYTGCATIGTGTCDSHIGG	2597-2578	
**VP4**	RC1	Sense	CCATATAGYTATTGGATTGGC	615-635	This study
	RC2M	Antisense	GGIARYTTCCACCACCAHCC	1197-1178	
**VP1-2A**	RC5M	Sense	TIACITTTGTSATHACIAG	2799-2817	This study
	RC6	Antisense	ACIACRCCYTCICCICCCAT	3690-3671	
	RC10	Antisense	TCCCACACRCAVYTYWGCC	3396-3378	This study
	RC5-B2	Sense	CTAACWTTYGTCATNACCAG	2813-2832	This study
	RC5-B4	Sense	CTYACMTTTGTSATYACYAG	2802-2821	This study
	RC5-B3	Sense	CTGACGTTTGTCATAACAAG	2798-2817	This study
	RC5(B5/B6)	Sense	GARYTVACYTTTGTSATHAC	2801-2820	This study
	RC11(B2,B4,B5,B6)	Antisense	TCYCTRTTRTARTCYTCCC	3417-3399	This study
	RC12(B3)	Antisense	TCYCTGTTGTAACTTTCCC	3411-3393	This study

***a*** The following standard ambiguity codes were used: D = G, A, or T; R = A or G; Y = T or C; N = A, T, C, or G; M = A or C; S = G or C; H = A, C or T; W = A or T and I = deoxyinosine.

***b*** With reference to the sequence of CV-B3.

### Template purification and sequencing

To determine the isolates’ nucleotide sequences and to identify those amino acid changes in the capsid proteins that could be associated with DAF dependent and independent phenotype, the amplicons were purified using the Montage PCR centrifugal filter devices (Millipore) or a Qiaquick gel extraction kit (QIAGEN, France), depending on the presence of single or multiple bands, respectively. The purified products of the P1 genomic region were sequenced using 10 pmol/μl of each primer and the GenomeLab Dye Terminator Cycle Sequencing Quick Start kit (Beckman Coulter, Villepinte, France) according to the manufacturer’s instructions. The electrophoresis and analysis of DNA sequence reactions were performed with the automated DNA sequencer CEQ8000 (Beckman Coulter, Villepinte, France). The obtained sequences for VP1, VP2, VP3, VP4 and VP1-2A which contain the main antigenic sites of neutralization and cellular attachment receptors were aligned using Clustal W (version 1.81) [32].

### Statistical analysis

Data were analyzed using Student's *t* test. P values < 0.05 were considered significant.

## Results

### Trans-Epithelial Electrical Resistance (TEER) measurement

**Figure 1:**
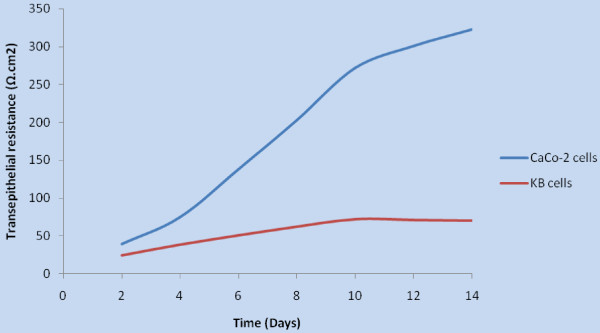
CaCo-2 Trans-Epithelial Electrical Resistance measurements before inoculation with CV-B isolates.

**Figure 2:**
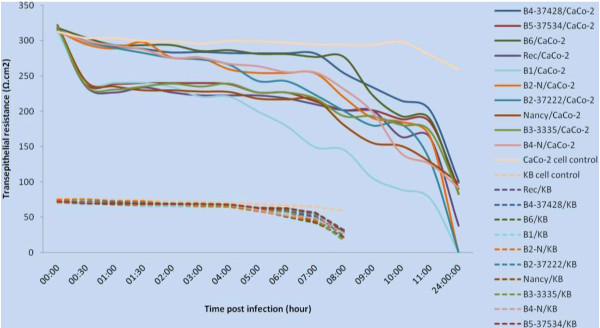
CaCo-2 and KB Trans-Epithelial Electrical Resistance measurements after inoculation with CV-B isolates.

Polarized monolayers of CaCo-2 cells were cultured in transwell filters until TEER measurement was stable (300 to 350 Ω.cm^2^ in 12 to 14 days) (Figure 1). The KB cells were tested in parallel. When the cells were exposed to virus isolates, a drop in TEER (Figure 2) was evident within 30 min pi for CV-B1, 3 and 5 serotypes. The same results were shown for the prototype strain Nancy. However, exposure of CaCo-2 monolayers to a viral isolate that binds CAR but not DAF (CV-B2, CV-B4 and CV-B6) had little or no effect on TEER. Interestingly, exposure to the recombinant isolate (Rec, CV-B3/B4) had the same effect as a CV-B3 isolate on monolayers of CaCo-2 cells. Within 7-11 h after infection, a drop in TEER was observed for all the tested isolates. Representative cytogram is depicted in Figure 2. Unlike polarized cells, the loss of membrane integrity in KB cells was not observed (Figure 2).

### Infection and inhibition assays

To further characterize the interaction of clinical isolates of CV-B with CAR/DAF, CV-B binding assays were performed on CaCo-2 cells coexpressing CAR and DAF. CV-B binding was assessed by MAb blockade of individual receptors or MAb blockade in combination. CaCo-2 cells infected in the absence of blocking antibodies were used as control (Table 2). All CV-B isolates exhibited high levels of cell lytic activity in the absence of blocking antibodies. Pre-treatment of Caco-2 cells with anti-CAR monoclonal antibody RmcB resulted in infection inhibition of all CV-B isolates. Virus titers (expressed in TCID_50_) in cultures treated with RmcB remained at a low level (Table 2) (p < 0.05). To determine the role of DAF during the virus infection, we used BRIC216, which has been shown to inhibit interaction between CV-B3 and DAF [33]. No inhibition of the replication of CV-B2 (CV-B2#N, CV-B2#37222, CV-B2#0610 M, CV-B2#2000), CV-B4 (CV-B4#N, CV-B4#37428, CV-B4#07, CV-B4#0612 and CV-B4-JVB) and CV-B6 (CV-B6#041) isolates was observed after pre-treatment of CaCo-2 cells with BRIC216. When the infectious titer of these isolates was measured, titers increased 48 h post infection to the level comparable with control cells (Table 2). These results imply that DAF is not necessary for the infection by these isolates. Virus attachment was however inhibited by BRIC216 for CV-B1 (CV-B1#032 and CV-B1# 0807), CV-B3 (CV-B3# 523–21 and CV-B3# 02) and CV-B5 (CV-B5 #37534 and CV-B5# 2003) isolates. The same result was obtained for the Nancy prototype strain. Similarly, adherence of Rec CV-B3/B4 isolate to CaCo-2 cells was significantly reduced in the presence of BRIC216 antibody. Productivity of infection was by 10^3^ and 10^4^-fold lower than measured on control cells (p < 0.05). Interestingly, 4 clinical CV-B isolates (CV-B3#37335, CV-B1#0609, CV-B5#99 and CV-B5#2000) were capable of lytically infecting the CaCo-2 cells in the presence of anti-DAF mAb. They were still able to efficiently replicate in Caco-2 cells, giving rise to the same amount of viral progeny than the control indicating an alternative attachment to Ca-Co2 cells. Finally, by combining anti-DAF and anti-CAR antibodies, viral attachment was significantly reduced and virus titers remained at a low level.

**Table 2:** **Virus titers (TCID**_50_*/50 μl) 48 h post infection without or in presence of anti-CAR (RmcB), anti-DAF (BRIC 216), or both

**Isolate**	**Control (No Ab)**	**+BRIC216**	**+ RcmB**	**RcmB + BRIC216**
Nancy	10^7.5^	10^3^	10^3^	10^2.5^
Rec (B3/B4)	10^8^	10^4^	10^3.7^	10^2^
B3-37335	10^7.3^	10^7^	10^3^	10^2^
B3-523-21	10^7.3^	10^3.6^	10^3.5^	10^2^
B3-02	10^6.6^	10^3.9^	10^3.3^	10^2^
B4N	10^7^	10^7^	10^4^	10^2^
B4-37428	10^6.6^	10^6^	10^3.3^	10^3^
B4 JVB	10^7.3^	10^7^	10^4^	10^2.7^
B4-0612	10^5.3^	10^5^	10^3.7^	10^2^
B4-07	10^5.3^	10^5^	10^3.5^	10^2^
B5-37534	10^7^	10^3^	10^3^	10^2^
B5-2003	10^8^	10^4.9^	10^3^	10^2^
B5-99	10^6.33^	10^6^	10^3^	10^2^
B5-2000	10^7.3^	10^7^	10^4^	10^2^
B6-041	10^5.9^	10^5^	10^3.5^	10^2^
B2-37222	10^6.3^	10^6^	10^4^	10^2.5^
B2N	10^6^	10^6^	10^3.5^	10^2.5^
B2-0610 M	10^5.3^	10^5^	10^3^	10^2^
B2-2000	10^6.3^	10^6^	10^4^	10^2.7^
B1-032	10^7.6^	10^3.9^	10^3^	10^3^
B1-0609	10^6.3^	10^6^	10^3^	10^2^
B1-0807	10^7.6^	10^4^	10^3^	10^2^

(*) TCID50: 50% Tissue Culture Infective Dose.

Ab: antibodies.

### Sequence analysis

Since CV-B isolates examined herein exhibited a strong CAR attachment- phenotype and for some isolates, either a DAF attachment phenotype or another unidentified phenotype, we speculate that they accumulated some amino acid differences. Comparison of the sequences of CV-B isolates revealed significant differences (Additional file 1). Previously, two residues 138D in VP2, and 234Q in VP3, had been identified as necessary for CV-B infection [34]. We have questioned if the attachment of these isolates in CAR-expressing cells is indeed due to the presence of these changes in amino acids and account for DAF attachment. We also tried to determine if there were perhaps other key residues associated with their phenotypes. Interestingly, we found that the Nancy strain has the two key residues associated with DAF-binding, the VP2-138 and the VP3-234. However, for the other isolates, there is DAF dependent isolates with “D” amino acid but also DAF independent isolates with the same amino acid in the position VP2-138. In addition, in the position VP3-234, we have DAF dependent isolates with “Q” and “D” amino acids versus DAF independent isolates with “Q”, “D” and “T” amino acids. In addition, when looking for other possible key residues that could be implicated in the DAF binding, we find that in position VP2-114, there’s the “K” amino acid (Lysin) for all the DAF dependent isolates in comparison with DAF independent isolates (S/H amino acids).

## Discussion and conclusions

Many viruses use multiple receptors to attach and enter susceptible target cells [16,35,36]. In addition, cellular receptor usage differs significantly between clinical isolates. Notably CV-B clinical isolates exhibit differences in their interactions with cell surface-expressed DAF [23]. DAF may sequester CV-B to the cell surface and maintain it in a conformationally unaltered state for subsequent interactions with CAR that induce capsid conformational changes and cell entry [24,37].

As a first step in the current study, the experiments were performed to assess the interactions of CV-B clinical isolates with DAF and CAR. Cell pre-treatment with RmcB resulted in a marked inhibition of all tested CV-B isolates. CaCo-2 infection by CV-B isolates was then clearly mediated by binding of the virus to CAR, to which Rmcb provided an effective blockade. All tested CV-B isolates show an absolute requirement for CAR but vary in their binding to DAF. The collected time points were titrated on KB cells (Table 2). The low titers obtained after 48 h are likely to be due to viral binding to regenerated CAR, no longer blocked by the antibody. These results support the virus usage of CAR when expressed on CaCo-2 cells, and indicate that binding to CAR is necessary to establish an efficient infection [23-26,28,38]. To determine the role of DAF during infection, we used BRIC216, which has been shown to inhibit interaction between CV-B3 and DAF [33]. The results implied that DAF was not necessary for CV-B2, CV-B4 and CV-B6 isolates. These viruses interact with the receptor (CAR), attach to and infect CaCo-2 cells. These results are in accordance with many previously published reports [12,23,38]. These isolates could also bind DAF with a lower affinity or bind another SRC than CV-B1, CV-B3 and CV-B5 DAF-binding strains. However, DAF was used by some isolates of CV-B1, CV-B3, and CV-B5. DAF was also used by the Nancy strain which is in contradiction with some studies [26,34]. A study of receptor preference in clinical CV-B1-5 isolates showed DAF binding viruses among CV-B1, 3 and 5, but not among the CV-B2 or CV-B4 isolates which have never been reported to use any other receptor but CAR. Interestingly, the Rec isolate (CV-B3/B4) was a DAF-dependent isolate behaving as a CV-B3 rather than a CV-B4 which raises the hypothesis that binding to DAF is the most adaptable way for infection. By molecular typing, the Rec isolate was identified as CV-B3 in the VP2 region and CV-B4 in the VP1 region, suggesting the occurrence of an interserotypic recombination event in the capsid region. The nucleotide sequence analysis of the whole structural genomic region showed the occurrence of a recombination event at position 1950 within the VP3 capsid gene, in a region coding for the 2b antigenic site previously described for CV-B3. Interestingly, the strain was neutralized by a polyclonal CV-B3-specific antiserum but not by a CV-B4-specific antiserum. The neutralization pattern suggests that the major antigenic site is located within the VP2 protein [39].

Taken together, these results suggest that even within a single serotype, viruses interact differently with cellular receptors. A synergistic inhibitory effect of anti-DAF and anti-CAR antibodies was observed with regard to infection which is in accordance with previous studies [40]. Antibody-mediated blockade showed that the presence of mAbs to both DAF and CAR was required to effect total inhibition of lytic infection.

In the present study, we also report the capacity of some clinical CV-B isolates not to utilize DAF interactions in a functional role. These isolates infect cells using either only CAR receptor or another co-receptor other than DAF. They may have adapted to utilize a novel as-yet-unidentified receptor present on CaCo-2 cells, thereby allowing virus attachment. If so, it is likely that the virus has only low affinity for this additional receptor, as most strains utilize DAF. A variant of CV-B3 has been shown to interact with heparan sulfate (HS) at the cellular surface [41]. HS has also been recognized as a receptor of many different viruses allowing initial binding to the cell surface including echoviruses, theilers murine encephalomyelitis virus, and foot-and-mouth disease virus [15,42,43]. HS seems to be a possible candidate that could be a co-receptor mediating the binding of these CV-B isolates to the cell surface. But, in cell entry, what is the role of virus binding to a likely low-affinity receptor? Some investigations are currently conducted into these matters. Receptor binding can vary among CV-B isolates, and may evolve within infected tissues or cultured cells [24]. Variants with altered co-receptor may arise spontaneously, either in the body or in tissue culture, and interaction with cells that do or do not express receptor molecules may exert powerful selective pressures on a virus population. Interaction between CV-B and their cell surface receptors appear then to be quite complex.

As a second step of the current study, the variation of the TEER of Caco-2 cells along with the viral growth cycle was followed at various times. Similarly to the results reported by Coyne and Bergelson (2006) [38], a drop in TEER (Figure 2) was evident within 30 min p.i for CV-B1, 3 and 5 serotypes and Rec isolate (CV-B3/B4) suggesting a transient partial loss of junctional integrity during early stages of virus entry, specifically when the virus had formed DAF clusters and was beginning to relocalize to the tight junctions (TJ). By binding to the CAR extracellular domain, CV-B may interfere with CAR-CAR interactions that are thought to contribute to junctional stability. Exposure of CaCo-2 monolayers to a viral isolate that binds CAR but not DAF (CV-B2, CV-B4 and CV-B6) had little or no effect on TEER, as reported by Shieh and Bergelson (2002) [13]_._ These viruses may avoid the barrier imposed by the tight junction by passing through M cells to reach submucosal lymphoid tissue. Within 7-11 h after infection, a drop in TEER was observed in all the tested isolates demonstrating the arrival to the final stages of the viral growth cycle and cell lysis (Figure 2). When polarized epithelial cells that express CAR in cell-cell junctions were found to be somewhat resistant to infection, but less resistant to CV-B that bind DAF as well as CAR, the relevance of DAF-binding CV-B strains was established [13,14].

Finally, in an attempt to gain a better understanding of the molecular basis of CV-B cellular interactions, the P1 region was sequenced and aligned. The data presented in this study estimated that in vitro selection of different phenotypes, may lead to the acquisition of amino acid mutations in the viral capsid proteins, thereby allowing the use of different and additional binding molecules. Surprisingly, some differences in the deduced amino acid sequences between clinical CV-B isolates were revealed (Additional file 1). Previously, Pan J et al. (2011) [34] produced cDNA clones encoding both CV-B3-RD and CV-B3-Nancy. They found that a single amino acid change, the replacement of a glutamate within VP3 (VP3-234E) with a glutamine residue derived from CV-B3-RD (VP3-234Q), confers upon CV-B3-Nancy the capacity to bind DAF and to infect RD cells. Furthermore, when CV-B3-H3—an isolate that does not bind to DAF although it already possesses VP3-234Q— was passed on RD cells, they obtained a DAF-binding isolate with a single amino acid change within VP2 (VP2-138 N to D). They conclude that both VP3-234Q and VP2-138D are required for virus attachment to DAF. In our study, we have questioned whether VP2-138D and VP3-234Q account for the CV-B phenotype DAF dependent (Additional file 1). In parallel, we tried to determine if there were perhaps other key residues associated with DAF binding. Interestingly, we found that the Nancy strain has the two key residues associated with DAF-binding, the VP2-138D and the VP3-234Q. However, for the other isolates, there is DAF dependent isolates with “D” amino acid but also DAF independent isolates with this amino acid. In addition, in the position VP3-234, we found DAF dependent isolates with “Q” and “D” amino acid versus DAF independent isolates with “Q”, “D” and “T” amino acids. Interestingly, when looking for other possible key residues that could be implicated in the DAF binding, we find that in position VP2-114 (Additional file 1), there’s a “K” amino acid (Lysin) common for all the DAF dependent isolates in comparison with DAF independent isolates (S/H amino acids). The structure of the CV-B-DAF complex determined by cryo-electron microscopy is then needed to confirm if VP2-114 K is in contact with DAF. When Lindberg et al. [44] constructed a series of genomic Chimeras between CV-B3-RD and a CV-B3-Nancy isolate (a strain that not binds DAF), they identified a genome segment that conferred the capacity to infect and kill RD cells efficiently—a phenotype that likely depends on, but is not necessarily synonymous with, DAF binding. Within this segment (which encodes VP2 and a part of VP3), the wild-type and RD isolates differed at two sites: in RD, a serine was substituted for threonine at position 151 within VP2 (VP2-151S) and a valine was substituted for aspartate at VP2 position 108 (VP2-108 V) but, neither VP2-151S nor VP2-108 V was found to be in contact with DAF.

In the light of these data, it is clear that multiple surface residues participate in binding DAF. So, the presence of 138D, 234Q or 114 K may contribute for DAF binding, but it does not suggest that they are necessary neither sufficient. Interaction between CV-B and their cell surface receptors appear then to be more complex. However, while not addressed in the present study, the involvement of additional changes located at other regions of the viral genome (e.g., 5_-untranslated region) cannot be ruled out. Further studies are presently underway to provide a better molecular understanding of theses phenotypes and determine the impact of receptor variation for infection and pathogenesis in vivo*.*

The present results indicate, moreover, that in some circumstances, DAF binding could not be used for some CVB isolates and to conclude, CV-B (at least CV-B1, 3 and 5) might be subdivided into two generalized DAF-binding phenotypes: CV-B that do not bind to DAF (which apparently includes most of all CV-B2, 4, and 6, as well as some isolates of CV-B1, 3 and 5) and CV-B that bind to DAF but require CAR for infection.

## Abbreviations

Ab: Antibody; CAR: Coxsackievirus adenovirus receptor; CV-B: Coxsackie B viruses; DAF: Decay accelerating factor; mAb: monoclonal antibody; pi: post infection; Rec: Recombinant isolate; SCR: Short consensus repeat.; TCID50: Tissue culture infectious dose 50%; TEER: Trans-Epithelial Electrical Resistance.

## Competing interests

The authors declare that they have no competing interests.

## Authors’ contributions

RS Carried out the molecular methods, the analysis and interpretation of these data and drafted the manuscript; HR carried out the TEER measurements; GI carried out the cell culture and helped to draft the manuscript; DN and SP carried out the conception of the primers; LB and SP participate in the alignment of sequences; PB and AM carried out the correction of the manuscript; PB is the director of this work and responsible for the general supervision of the research group. All authors read and approved the final manuscript.

## Supplementary Material

Additional file 1 **The solid vertical lines delineate the four structural viral proteins (VP).** The antigenic sites described for CV-B3 by Auvinen et al. [45] are depicted in gray line and designated according to the nomenclature of those authors. The asterisks design sequence homologies, the signs «. » and “:” design amino acid differences. Regarding positions exhibiting amino acid differences or homologies between the two phenotypes, blue vertical line locates CV-B surface residues predicted to interact with DAF, VP2-138 and VP3-234. Yellow vertical line locates other CVB surface residues that could interact with DAF, VP2-114 K. The amino acids are numbered according to the sequence of the reference strain of CV-B3 (accession number M16572). The positions of the main tertiary structures described for CV-B3 [46] are indicated by double-headed horizontal arrows above the sequences. Sequence alignments were generated by using the Clustal W (version 1.81) program [32]. Click here for file
