# Supplementary material for: Study of Coxsackie B viruses interactions with Coxsackie Adenovirus receptor and Decay-Accelerating Factor using Human CaCo-2 cell line
Source: J Biomed Sci. 2014 May 21;21(1):50. doi: 10.1186/1423-0127-21-50 (PMC4035751; doi:10.1186/1423-0127-21-50)
Supplement: Additional file 1 — The solid vertical lines delineate the four structural viral proteins (VP). The antigenic sites described for CV-B3 by Auvinen et al. [45] are depicted in gray line and designated according to the nomenclature of those authors. The asterisks design sequence homologies, the signs «. » and “:” design amino acid differences. Regarding positions exhibiting amino acid differences or homologies between the two phenotypes, blue vertical line locates CV-B surface residues predicted to interact with DAF, VP2-138 and VP3-234. Yellow vertical line locates other CVB surface residues that could interact with DAF, VP2-114 K. The amino acids are numbered according to the sequence of the reference strain of CV-B3 (accession number M16572). The positions of the main tertiary structures described for CV-B3 [46] are indicated by double-headed horizontal arrows above the sequences. Sequence alignments were generated by using the Clustal W (version 1.81) program [32]. [file 1423-0127-21-50-S1.pdf]

**Figure 3**

```
1           VP4                                                                   70 | VP2
B3-02       MGAQVSTQKTGAHETGVSASGNSIIHYTNINYYKDAASNSATRQDFAQDPGKFTEPVKDIMIKSLPALNSPTVEECGYSDRVRSITLGNSTITTQECANVVVGYGVWPDYLKDDEATAED
B3-523-21   MGAQVSTQKTGAHETGVNASGNSIIHYTNINYYKDAASNSATRQDFAQDPGKFTEPVKDIMIKSLPALNSPTVEECGYSDRVRSITLGNSTITTQECANVVVGYGVWPDYLKDDEATAED
B5-37-534   ------MXKTGAHETGLNASGNSIIHYTNINYYKDAASNSANRQEFAQDPGKFTEPVKDIMIKSMPALNSPSAEECGYSDRVRSITLGNSTITTQECANVVVGYGTWPTYLKDEEATAED
B1-032      MGAQVSTQKTGAHETGLNASGNSIIHYTNINYYKDAASNSANRQDFTQDPGKFTEPVKDIMIKSMPALNSPSAEECGYSDRVRSITLGNSTITTQECANVVVGYGVWPEYLKDNEATAED
B1-0807     MGAQVSTQKTGAHETGLNASGNSIIHYTNINYYKDAASNSANRQDFTQDPGKFTEPVKDIMIKSMPALNSPSAEECGYSDRVRSITLGNSTITTQECANVVVGYGVWPEYLKDNEATAED
REC-B3/B4   MGAQVSTQKTGAHETGLNASGNSIIHYTNINYYKDAASNSANRQDFAQDPGKFTEPVKDIMIKSLPALNSPTVEECGYSDRVRSITLGNSTITTQECANVVVGYGVWPDYLKDDEATAED
CV-B4-JVB   MGAQVSTQKTGAHETSLSASGNSIIHYTNINYYKDAASNSANRQDFTQDPSKFTEPVKDVMIKSLPALNSPTVEECGYSDRVRSITLGNSTITTQECANVVVGYGVWPDYLSDEEATAED
B4-07       MGAQVSTQKTGAHETNLSASGNSIIHYTNINYYKDAASNSANRQDFAQDPSKFTEPVKDVMIKSLPALNSPTVEECGYSDRVRSITLGNSTITTQECANVVVGYGVWPDYLSDEEATAED
B4-37-428   MGTQVSTQKTGAHETSLKASGNSIIHYTNINYYKDAASNSANRQDFTQDPSKFTEPVKDVMIKSLPALNSPTVEECGYSDRVRSITLGNSTITTQECANVVVGYGVWPDYLSDEEATAED
CV-B3-Nancy MGAQVSTQKTGAHETRLNASGNSIIHYTNINYYKDAASNSANRQDFTQDPGKFTEPVKDIMIKSLPALNSPTVEECGYSDRARSITLGNSTITTQECANVVVGYGVWPDYLKDSEATAED
B6-041      MGAQVSTQKTGAHETALNAQGNSVIHYTNINYYKDAASNSANRQDFTQDPSKFTEPVKDVMIKSLPALNSPTVEECGYSDRVRSITLGNSTITTQECANVVVAYGVWPDYLHDDEATAED
B2-37-222   MGAQVSTQKTGAHETGLSASGGSIIHYTNINYYKDAASNSANRQDFTQDPGKFTEPVKDIMIKSMPALNSPSAEECGYSDRVRSITLGNSTITTQECANVVVGYGTWPRYLSDKEATAED
                    ******* :.*.*.*:*****************.**:*:***.********:****:****:******:.********.********************.**.** ** *.******

                                                                                        EF loop (PUF)          site 2b
121
B3-02       QPTQPDVATCRFYTLDSVQWQKTSPGWWWKLPDALSNLGLFGQNMQYHYLGRTGYTIHVQCNASKFHQGCLLVVCVPEAEMGCATLDNTPSSEELLGGDAAKEFADKP-VASGSNKLVQR
B3-523-21   QPTQPDVATCRFYTLDSVQWQKTSPGWWWKLPDALSNLGLFGQNMQYHYLGRTGYTIHVQCNASKFHQGCLLVVCVPEAEMGCATLDNTPSSEELLGGDAAKEFADKP-VASGSNKLVQR
B5-37-534   QPTQPDVATCRFYTLESVMWQQSSPGWWWKFPDALSNMGLFGQNMQYHYLGRAGYTVHVQCNASKFHQGCLLVVCVPEAEMGCATLANKPDQKSLSNGETANTFDSQN-TTGQT--AVQA
B1-032      QPTQPDVATCRFYTLESVQWMKNSAGWWWKLPDALSQMGLFGQNMQYHYLGRTGYTIHVQCNASKFHQGCLLVVCVPEAEMGCSNLNNTPEFAELSGGDNARMFTDTE-VGTSNNKKVQT
B1-0807     QPTQPDVATCRFYTLESVQWMKNSAGWWWKLPDALSQMGLFGQNMQYHYLGRTGYTIHVQCNASRFHQGCLLVVCVPEAEMGCSNLNNTPEFAELSGGDVAKVFTDTK-VGESNAKKVQT
REC-B3/B4   QPTQPDVATCRFYTLDSVQWQKTSPGWWWKLPDALSNLGLFGQNMQYHYLGRTGYTIHVQCNASKFHQGCLLVVCVPEAEMGCATLDNTPSSEELLGGDAAKEFADKP-VASGSNKLVQR
CV-B4-JVB   QPTQPDVATCRFYTLNSVKWEMQSAGWWWKFPDALSEMGLFGQNMQYHYLGRSGYTIHVQCNASKFHQGCLLVVCVPEAEMGCTNAENAPAYGDLCGGETAKSFEQN--AATG-KTAVQT
B4-07-final QPTQPDVATCRFYTLNSVKWEMQSAGWWWKFPDALSEMGLFGQNMQYHYLGRSGYTIHVQCNASKFHQGCLLVVCVPEAEMGCTNAENAPTYGDLCGGETAKQFEQN--AATG-ETAVQT
B4-37-428   QPTQPDVATCRFYTLNSVKWEMQSAGWWWKFPDALSEMGLFGQNMQYHYLGRSGYTIHVQCNASKFHQGCLLVVCVPEAEMGCTNAENAPTYDDLCGGETAKQFEQN--TATG-KTAVQT
CV-B3-Nancy QPTQPDVATCRFYTLDSVQWQKTSPGWWWKLPDALSNLGLFGQNMQYHYLGRTGYTVHVQCNASKFHQGCLLVVCVPEAEMGCATLDNTPSSAELLGGDTAKEFADKP-VASGSNKLVQR
B6-041      QPTQPDVATCRFYTLDSVSWQSSSAGWWWKFPDALSNMGLFGQNMQYHYLGRSGYTIHVQCNASKFHQGCLLVVCVPEAEMGCSNLNNAPLAADLSAGEVARQFTVEP-ANGQN--QVQX
B2-37-222   QPTQPDVATCRFYTLSSVQWQRESAGWWWKFPDALSDMGLFAQNMMYHYLGRTGYTIHVQCNASKFHQGCLLVVCVPEAEMGCTNKENTPLFENLCGQDNAKEFTREGPTISKGATDVQT
            ***************.** *    *.*****:*****::***.*** ******:***:*******:******************:.  * *    .*    : *. *        .        **

                                                                                                          330 | VP3
242
B3-02       VVYNAGMGIGVGX----------------XLTIMPYTNSVPMDNMFIHKNVTLMVIPFVPLDYCPGSTTYVPITVTIAPMCTEYNGLRLAGHQGLPTMNTPGSCQFLTSDDFQSPSAMPQ
B3-523-21   VVYNAGMGIGVGX---------------XATIVMPYTNSVPMDNMFRHKNVTLMVIPFVPLDYCPGSTTYVPITVTIAPMCAEYNGLRLAGHQGLPTMNTPGSCQFLTSDDFQSPSAMPQ
B5-37-534   NVINAGMGVGVGNITIX--------------IVMPYINSVPVDNMFRHNICTLMIIPFAPRSYSTGATTYVPITVTVAPMCAEYNGLRLAGKQGLPTMLTPGSNQFLTSDDFQSPSAMPQ
B1-032      AVWNAGMGVGVGNLTIXWI---NLRTNNSATIVMPYINSVPMDNMYRHNNLTLMIIPFVPLNYSEGSSPYVPITVTIAPMCAEYNGLRLASSQGLPVMTTPGSTQFLTSDDFQSPSAMPQ
B1-0807     AVWNAGMGVGVGNLTIFX-------------IVMPYINSVPMDNMFRHNNITLMIIPFVPLNYSEGSSPYVPITVTIAPMCAEYNGLRLASNQGLPVMTTPGSTQFLTSDDFQSPSAMPQ
REC-B3/B4   VVYNAGMGIGVGNLTIFPHQWINLRTNNSATIVMPYTNSVPMDNMFRHNNVTLMVIPFVPLDYCPGSTTYVPITVTIAPMCAEYNGLRLAGHQGLPTMNTPGSCQFLTSDDFQSPSAMPQ
CV-B4-JVB   AVCNAGMGVGVGNLTIYPHQWINLRTNNSATIVMPYINSVPMDNMFRHNNFTLMIIPFAPLDYVTGASSYIPITVTVAPMSAEYNGLRLAGHQGLPTMLTPGSTQFLTSDDFQSPSAMPQ
B4-07       AVCNAGMGVGVGNLTIYX------RTNNSATIVMPYINSVPMDNMFRHNNFTLMIIPFAPLDYVAGASSYIPITVTVAPMSAEYNGLRLAGHQGLPTMLTPGSTQFLTSDDFQSPSAMPQ
B4-37-428   AVCNAGMGVGVG-------------------XVMPYINSVPMDNMYRHNNFTLMIIPFAPLDYCPGSTTYVPITVTIAPMCAEYNGLRLAGHQGLPTMNTPGSCQFLTSDDFQSPSAMPQ
CV-B3-Nancy VVYNAGMGVGVGNLTIFPHQWINLRTNNSATIVMPYTNSVPMDNMFRHNNVTLMVIPFVPLDYVTGASSYIPITVTVAPMSAEYNGLRLAGHQGLPTMLTPGSTQFLTSDDFQSPSAMPQ
B6-041      ----------XTNNSAT--------------IVMPYINSVPMDNMFRHNNFTLMIIPFAKLAYSDGASTFVPITVTIAPMNAEYNGLRLAGHQGLPVMTTPGSTQFLTSDDFQSPCAMPQ
B2-37-222   AVCNAGMGVXXAT------------------IVMPYINSVPMDSMIRHNNFTLMIIPFVPLDYVNGSSPYIPITVTVAPMSAEYNGLRLASTQGLPTMLTPGSNQFLTSDDFQSPSAMPQ
                                           :*** ****:*.*  *:  ***:***.   *   *::.::*****:*** :********. ****.* **** ***********.****
```

```
                                                   site 3b
                                   Knob          BC  loop
                                 <------>   |    <--->
363
B3-02          YDVTPEMRIPGEVKNLMEIAEVDSVVPVQNIGEKVNSMEAYQIPVRSNEGTGTQVFGFPLQPGYSSVFSRTLLGEILNYYTHWSGSIKLTFMFCGSAMATGKFLLAYSPPGAGAPTKRVD
B3-523-21      YDVTPEMRIPGEVKNLMEIAEVDSVVPVQNIGEKVNSMEAYQIPVRSNEGTGTQVFGFPLQPGYSSVFSRTLLGEILNYYTHWSGSIKLTFMFCGSAMATGIFLLAYSPPGAGAPTKRVD
B5-37-534      FDVTPEMAIPGQVNNLMEIAEVDSVVPVNNIEGKVSSIEAYQIPVQSNSTNGSQVFGFPLIPGASSVLNRTLLGEILNYYTHWSGSIKLTFMFCGSAMATGKFLLAYSPPGAGAPTTRKE
B1-032         FDVTPEMQIPGRVNNLMEIAEVDSVVPVNNTDTNVNGLKAYQIPVQSNSDNGRQVFGFPLQPGANNVLNRTLLGEILNYYTHWSGSIKLTFMFCGSAMATGKFLLAYSPPGAGVPKNRRD
B1-0807        FDVTPEMQIPGRVNNLMEIAEVDSVVPVNNTEDNVSSLKAYQIPVQSNSDNGKQVFGFPLRPGANNMSNRALLGX---------------------------FLLAYSPPGAGVPKNRRD
REC-B3/B4      YDVTPEMRIPGEVKNLMEIAEVDSVVPVQNIGEKVNSMEAYQIQVRSTDEMGGQIFGFPLQPGASSVLQRTLLGEILNYYTHWSGSLKLTFVFCGSAMATGKFLLAYSPPGAGAPDSRKN
CV-B4-JVB      FDVTPEMNIPGQVRNLMEIAEVDSVVPLNNLKANLTTMEAYRVQVRSTDEMGGQIFGFPLQPGASSVLQRTLLGEILNYYTHWSGSLKLTFVFCGSAMATGKFLLAYSPPGAGAPDSRKN
B4-07          FDVTPEMNIPGQVRNLMEIAEVDSVVPINNLKANLKTMEAYRVQVRSTDEMGGQIFGFPLQPGASSVLQRTLLGEILNYYTHWSGSLKLTFVFCGSAMATGKFLLAYSPPGAGAPDSRKN
B4-37-428      YDVTPEMRIPGEVKNLMEIAEVDSVVPVQNIGEKVNSMEAYRVQVRSTDEMGGQIYGSPLQPGASSVLQRTLLGEILNYYTHWSGSLKLTFVFCGSAMATGKFLLAYSPPGAGAPDSRKN
CV-B3-Nancy    FDVTPEMNIPGQVRNLMEIAEVDSVVPINNLKANLMTMEAYQIPVRSNEGSGTQVFGFPLQPGYSSVFSRTLLGEILNYYTHWSGSIKLTFMFCGSAMATGKFLLAYSPPGAGAPTKRVD
B6-041         FDVTPEMNIPGQVNNLMEIAEVDSVVPVNNTETNVNGMDAYRIPVQSNMDTGGQVFGFPLQPGASSVFQRTLLGEILNYYTHWSGSIKLTFMFCGSAMATGKFLLAYSPPGAGAPKSRKD
B2-37-222      FDVTPEMNIPGRVHNLMEIAEVDSVVPLNNIQDNLRKMDIYRVQVSSQTSQGAQVFGFSLQPGASSVLQRTLLGX-----------------------TGKFLLAYSPPGAGVPPDRKK
               :****** ***.*.*************::*    ::  :.  *::  *  *     *  *::*  .*  **   ..:  .*:***                            ***********.*  *

                                                                                                                    VP1
484                                                                                       580
B3-02          AMLGTHVVWDVGWQSSCVLCIPWISQTHYRYVASDEYTAGGFITCWYQTNIVVPADAQSSCYIMCFVSACNDFSVSILKDTPFISQQNFLQGPVEDAVTAAXX-----------------
B3-523-21      AMLGTHVVWDVGLQSSCVLCIPWISQTHYRYVASDEYTAGGFITCWYQTNIVVPADAQSSCYIMCFVSACNDFSVSILKDTPFISQX--------------X-----------------
B5-37-534      AMLGTHVIWDVGLQSSCVLCIPWISQTHYRYVVVDEYTAGGYITCWYQTNIVVPADTQSDCKILCFVSACNDFSVRMLNDTLFIKQDSFFQGPPGEAVERAIARVADTIT----------
B1-032         AMLGTHVIWDVGLQSSCVLCVPWISQTHYRYVVEDEYTAAGYVTCWYQTNIIVPADVQSTCDILCFVSACNDFSVRMLKDTPFIRQDNFYQGPVEESVARAMVRVADTVSXX--------
B1-0807        AMLGTHVIWDVGLQSSCVLCVPWISQTHYRYVVEDEYTAAGYVTCWYQTYIIVPADVQSTCDILCFVSACNDFSVRMLKDTPFIRQDNFYQGPVEESVERAMVRVADTVSSX--------
REC-B3/B4      AMLGTHVIWDVGLQSSCVLCVPWISQTHYRYVVDDKYTASGFISCWYQTNVIVPAEAQKSCYIMCFVSACNDFSVRMLRDTQFIKQDNFYQGPTEDSVERAMGRVADTIARGPSNSEQIP
CV-B4-JVB      AMLGTHVIWDVGLQSSCVLCVPWISQTHYRYVVDDKYTASGFISCWYQTNVIVPAEAQKSCYIMCFVSACNDFSVRMLRDTQFIKQTNFYQGPTEESVERAMGRVADTIARGPSNSEQIP
B4-07          AMLGTHVIWDVGLQSSCVLCVPWISQTHYRYVVDDKYTASGFISCWYQTNVIVPAEAQKSCYIMCFVSACNDFSVRMLRDTQFIKQDNFYQGPTEDAVEPAMGRVADTIARGPSNSE---
B4-37-428      AMLGTHVIWDVGLQSSCVLCVPWISQTHYRYVVDDKYTASGFISCWYQTNVIVPAEAQKSCYIMCFVSACNDFSVRMLRDTQFIKQDNFYQGPTEESVERAMGRVADTIAX---------
CV-B3-Nancy    AMLGTHVIWDVGLQSSCVLCIPWISQTHYRFVASDEYTAGGFITCWYQTNIVVPADAQSSCYIMCFVSACNDFSVRLLKDTPFISQQNFFQGPVEDAITAAIGRVADTVGTGPTNSEAIP
B6-041         AMLGTHVIWDVGLQSSCVLCIPWISQTHYRFVVADEYTAGGFITCWYQTNVIVPLGAQSNCSILCFVSACNDFSVRMLRDTKFISQTAFYQSPVEGAIERAIARXXAVET----------
B2-37-222      AMLGTHVIWDVGLQSSCVLCVPWISQTHYRYTVKDEYTDSGYITCWYQTNVIAPADALSTCYIMCMVSACNDFSVRMLRDTRFIKQTAFYQSPVEESIERSIGRVADTIGSG--------
               *******:**** *******:*********:..  *:**  .*:::*****  ::.*    .  .   *  *:*:*********  :*.** ** *

                                                                 site 1
                                                                   |  BC  loop
                                                                      <------->
605
B3-02          ---AAETGHTSQVVPGDTMQTRHVKNYHSRSESTIENFVCRSACVYFTEYENS-GSNR---YAEWVITTRQAVQLRRKLEFFTYMRFDLELTFVITSTQQPSTTQNQDAQILTHQIMYVP
B3-523-21      ---AVETGHTSQVIPGDTMQTRHVKNYHSRSESTIENFVCRSACVYFTEYENS-GSNR---YAEWVITTRQAVQLRRKLEFFTYMRFDLELTFVITSTQQPSTTQNQDAQILTHQIMYVP
B5-37-534      -------GHTSQVVPADTMQTRHVKNYHSRSESTVENFLCRSACVYYTTYKNH-GTDGDN-FAYWVINTRQVAQLRRKLEMFTYARFDLELTFVITSTQEQSTIQGQDSPVLTHQIMYVP
B1-032         ---AAETGHTSQVVPSDTMQTRHVKNYHSRSESSIENFLCRSACVYYATYTNN-SKKGY---AEWVINTRQAAQLRRKLELFTYLRFDLELTLVITSAQQPSTATSVDAPVQTHQIMYVP
B1-0807        ---AAETGHTSQVVPSDTMQTRHVKNYHSRSESSVENFLCRSACVYYATYTNN-TEKG---YAEWVVNTRQVAQLRRKLELFTYLRFDLELTFVITSAQQPSTATSVDAPVQTHQIMYVP
REC-B3/B4      ALTAVETGHTSQVDPSDTMQTRHVHNYHTRSESSIENFLCRSACVIYIKYSSA-ESNNLKRYAEWVINTRQVAQLRRKMEMFTYIRCDMELTFVITSHQEMSTATNSDVPVQTHQIMYVP
CV-B4-JVB      ALTAVETGHTSQVDPSDTMQTRHVHNYHSRSESSIENFLCRSACVIYIKYSSA-ESNNLKRYAEWVINTRQVAQLRRKMEMFTYIRCDMELTFVITSHQEMSTATNSDVPVQTHQIMYVP
B4-07          ---XVETGHTSQVDPSDTMQTRHVHNYHSRSESSIENFLCRSACVIYIKYSSA-ESNNLKRYAEWVINTRQVAQLRRKMEMFTYIRCDMELTFVITSHQEMSTATNSDVPVQTHQIMYVP
B4-37-428      ----VETGHTSQVDPSDTMQTRHVHNYHSRSESSIENFLCRSACVIYIKYSSA-ESNKLKRYAEWVINTRQVAQLRRKMEMFTYIRCDMELTFVITSHQEMSAATNSDVPVQTHQIMYVP
CV-B3-Nancy    ALTAAETGHTSQVVPGDTMQTRHVKNYHSRSESTIENFLCRSACVYFTEYKNS-GAKR---YAEWVLTPRQAAQLRRKLEFFTYVRFDLELTFVITSTQQPSTTQNQDAQILTHQIMYVP
B6-041         -------GHTSQVVPSDNMQTRHVKNYHSRSETSVENFLCRSACVYFTTYKNQ-TGAKNR-FASWVITTRQVAQLRRKLEMFTYLRFDIELTFVITSAQDQSTIS-QDAPVQTHQIMYVP
B2-37-222      ----PSTGHTSQVTPSDTMQTRHVHNYHSRSESSVENFLARSACVFYTTYTNSKNAAKEKKFATWKVSVRQAAQLRRKLELFTYLRCDIELTFVITSAQDPSTATNLDVPVLTHQIMYVP
                      ****** *.*.******:***:***:::***:.*****  :   *  .         :*  *  :.  **..*****:*:***  *  *:***:****  *:  *:      *   :  ********
```

```
                                                       site 2a ↓
726
B3-02           PGGPVPDKVDSYVWQTSTNPSVFWTEGNAPPRMSIPFLSIGNAYSNFYDGWSEFARSGVYGINTLNNMGTLYARHVNAGSTGPIKSTIRIYFKPKHVKAWIPRPPRLCQYEKAKNVNFQP
B3-523-21       PGGPVPDKVDSYVWQTSTNPSVFWTEGNAPPRMSIPFLSIGNAYSNFYDGWSEFARSGVYGINTLNNMGTLYARHVNAGSTGPIKSTIRIYFKPKHVKAWIPRPPRLCQYEKAKNVNFQP
B5-37-534       PGGPVPTKINSYSWQTSTNPSVFWTEGSAPPRISIPFISIGNAYSMFYDGWARFDKQGTYGINTLNNMGTLYMRHVNDGSPGPIVSTVRIYFKPKHVKTWVPRPPRLCQYQKAGNVNFEP
B1-032          PGGPVPTKVTDYAWQTSTNPSVFWTEGNAPPRMSIPFISIGNAYSCFYDGWTQFSRNGVYGINTLNNMGTLYVRHVNEAGQGPIKSTVRIYFKPKHVTAWVPRPPRLCQYEKQKNVNFNP
B1-0807         PGGPVPTKVTDYAWQTSTNPSVFWTEGNAPPRMSIPFISIGNAYSCFYDGWTQFSRNGVYGINTLNNMGTLYMRHVNEAGQGPIKSTVRIYFKPKHVKAWVPRPPRLCQYEKQKNVNFNP
REC-B3/B4       PGGPVPTSVNDYVWQTSTNPSIFWTEGNAPPRMSIPFMSIGNAYTMFYDGWSNFSRDGIYGYNSLNNMGTIYARHVNDSSPGGLTSTIRIYFKPKHVKAYVPRPPRLCQYKKAKNVNFDV
CV-B4-JVB       PGGPVPTSVNDYVWQTSTNPSIFWTEGNAPPRMSIPFMSIGNAYTMFYDGWSNFSRDGIYGYNSLNNMGTIYARHVNDSSPGGLTSTIRIYFKPKHVKAYVPRPPRLCQYKKAKSVNFDV
B4-07           PGGPVPTSVNDYVWQTSTNPSIFWTEGDAPPRMSIPFMSIGNAYTMFYDGWSNFSRDGIYGYNSLNNMGTIYARHVNDSSPGGLTSTIRIYFKPKHVKAYVPRPPRLCQYKKAKNVNFDV
B4-37-428       PGGPIPTSVHDYVWQTSTNPSIFWTEGNAPPRMSIPFMSIGNAYTMFYDGWSNFSRDGIYGYNSLNNMGTIYARHVNDSSPGGLTSTIRIYFKPKHVKAYVPRPPRLCQYKKAKNVNFDV
CV-B3-Nancy     PGGPVPDKVDSYVWQTSTNPSVFWTEGNAPPRMSIPFLSIGNAYSNFYDGWSEFSRNGVYGINTLNNMGTLYARHVNAGSTGPIKSTIRIYFKPKHVKAWIPRPPRLCQYEKAKNVNFQP
B6-041          PGGPVPTKVDDYAWQTSTNPSVFWTEGNAPPRMSVPFMSIGNAYSTFYDGWSDFSNKGIYGLNTLNNMGTLYIRHVNGPNPIPITSTVRIYFKPKHVKAWVPRPPRLCQYKTSRQVNFTV
B2-37-222       PGGPVPETVDDYNWQTSTNPSLFWTEGNAPPRMSIPFMSIGNAYSMFYDGWSEFRHDGVYGLNTLNNMGTIYARHVNADNPGSITSTVRIYFKPKHVKAWIPRPPRLAQYLKANNVNFKI
                ****:* .: .* ********:*****.****:*:**:******: *****: * ..* ** *:******:* ****  .   : **:*********.:::******.** .  .***

847
B3-02           SGVTTTRQSITAMTNT
B3-523-21       SGVTTTRQSITAMTNT
B5-37-534       TGVTESRTEITAMQTT
B1-032          TGVTTTRSNITTT---
B1-0807         TGVTTSRLDITTT---
REC-B3/B4       EAVTTERANLVTT---
CV-B4-JVB       EAVTAERASLITT---
B4-07-final     EAVTTERANLVTT---
B4-37-428       EAVTTKRASLITT---
CV-B3-Nancy     SGVTTTRQSITTMTNT
B6-041          TGVTESRANITTMNTT
B2-37-222       TNVTEKRDSLTTT---
                **  * .: :
```
